# Mechanisms of Resilience in Children of Mothers Who Self-Report with Depressive Symptoms in the First Postnatal Year

**DOI:** 10.1371/journal.pone.0142898

**Published:** 2015-11-30

**Authors:** Emily Savage-McGlynn, Maggie Redshaw, Jon Heron, Alan Stein, Maria A. Quigley, Jonathan Evans, Paul Ramchandani, Ron Gray

**Affiliations:** 1 Policy Research Unit in Maternal Health and Care, National Perinatal Epidemiology Unit, University of Oxford, Oxford, United Kingdom; 2 School of Social and Community Medicine, Faculty of Medicine and Dentistry, University of Bristol, Bristol, United Kingdom; 3 Department of Psychiatry, University of Oxford, Oxford, United Kingdom; 4 Centre for Academic Mental Health, University of Bristol, Bristol, United Kingdom; 5 Department of Medicine, Imperial College London, London, United Kingdom; Maastricht University, NETHERLANDS

## Abstract

**Background:**

Symptoms of maternal postnatal depression are associated with an increased risk of adverse effects on child development. However, some children exposed to postnatal depression have outcomes similar to unexposed children, and can be referred to as resilient. This study aimed to determine the mechanisms of resilience in children exposed to depressive symptoms postnatally.

**Method:**

Data are from a prospective cohort study, the Avon Longitudinal Study of Parents and Children. Self-report questionnaire data were collected during pregnancy and the child’s first 2 years regarding maternal views of parenting and her perception of the child. The Edinburgh Postnatal Depression Scale (EPDS) was completed postnatally at 8 months and the Strengths and Difficulties Questionnaire (SDQ) at age 11 years. Exposed children who scored above the median score of non-exposed children were defined as resilient. Structural equation modeling was used to investigate the development of resilience.

**Results:**

From the core ALSPAC cohort, 1,009 children (6.9%) were exposed to maternal depression at 8 months postnatally. The SDQ total difficulties scores at 11 years of age indicated that 325 (32.2%) were resilient, 684 were non-resilient. Maternal positive feelings about parenting and child non-verbal communication at 15 months increased the likelihood of later resilience.

**Conclusions:**

In this study, resilience was associated with two factors: the child’s nonverbal communication at 15 months and by maternal positive feelings about parenting. Early intervention to support mother-child interaction and foster child development in women identified with postnatal depressive symptoms may benefit later child resilience.

## Introduction

Depression is one of the most prevalent conditions that women face after the birth of their child. In the general population, approximately 10–15% of mother will suffer from the condition [1,2], although this prevalence is considered by some to be underestimated [3]. Evidence suggests that infants whose mother experiences postnatal depression are at increased risk of adverse developmental outcomes in the child’s emotional, social, and cognitive development [4,5]. Consequently, maternal depression in the postnatal period can be viewed as an important public health concern due to the high prevalence of the disorder and the negative consequences facing both mother and child [6]. However, of the children exposed to postnatal depression in infancy, many will achieve developmental outcomes similar to their peers who were not exposed. By better understanding the underlying mechanisms by which such children show later resilience, there may be opportunities to learn more about ways of intervening and preventing adverse outcomes from the effects of postnatal depression.

One of the hypothesized mechanisms of the transmission of the adverse effect and a potential source for intervention is through parenting behaviors and cognitions relating to parenting roles. In infancy, maternal postnatal depression can interfere with specific aspects of maternal parenting capacities which can adversely affect the interaction between mother and infant, and ultimately, affect the child’s emotional and behavioral development [7,8]. More specifically, it is thought that depression disrupts maternal engagement and the mother’s ability to provide warm, consistent and sensitive parenting to her infant with increased levels of negative maternal affect, hostility, and withdrawal [9,10,11]. During early infancy, children are highly sensitive to the interpersonal environment that surrounds them [12]. Emotionally healthy mothers facilitate dyadic interactions, by sensitively and attentively responding to the child, helping them to regulate their arousal states. In doing so, the mother plays an important role in the development of her child’s emotional and behavioral regulation [13]. This is often framed within the context of attachment styles, particularly when associated with secure attachment. It should be noted that within the dyad, the nature of the interaction is not unidirectional, and that the child also contributes a great deal to the interactions that occur [12].

When suffering from depression, the mother’s ability to be available to her child and respond in an appropriately sensitive and timely manner necessary to facilitate this behavioral learning may be impaired through such mechanisms as rumination or perceived poorer self-efficacy as a mother [14,8]. Mothers who feel less effective as parents tend to have greater difficulty establishing warm and harmonious relationships with their baby [15], which in turn, may have an effect on the quality of attachment. Over time, there is an increased likelihood that the interrupted interactions between mother and child affect the child’s capacity for emotional regulation and understanding predictability of response [13], thereby influencing their learned responses when interacting with others. Due to the effects of postnatal depression, disturbances to child development may be evident in both the short- and the long-term and may persist long after the mothers’ condition has remitted [16,17]. By 36 months of age, such children are found to be five times more insecurely attached to their primary caregiver, display significantly more problem behavior, and perform more poorly on measures of cognitive development [18,19,20]. Around five years of age, the poor performance on cognitive measures continues, and children are exhibiting more social and emotional difficulties, including difficulties with self-control and increased depressed thinking [18]. By sixteen years of age, children are significantly more likely to be diagnosed with depression themselves [17].

However, not all children who have been exposed to adverse life circumstance, such as maternal depression, suffer from negative outcomes. Some children are resilient to the adversity they have faced and are able to achieve normative developmental outcomes relative to their peers [21], [22]’ [23].

Resilience can be thought of as a 2-dimensional construct inferred from measures of adversity and positive adaptation (or the absence of emotional or behavioral maladjustment) rather than a personality trait or an individual attribute [24,25]. Many factors have been suggested to support the development of resilience, such as individual characteristics, the family, as well as environmental aspects [26], yet none so strongly as the pivotal role of the parents.

Parental factors have been shown to promote coping capacities that enable children to do well in life in the face of difficulty such as positive parent-child relationships [27], high-quality parent-infant interaction [28] and secure parent-child attachment [29,30]. Further, high and consistent role models; harmony between parents; spending time with children; and provision of structure and rules have been found to serve a protective role in the face of adversity. Despite suffering from depressive symptoms, it may be possible for mothers to moderate their mood and responses towards their child, as a function of their parenting perspectives and behavior [31,32]. In so doing, this may help children to develop intrinsic resilient capacities and also to mediate coping responses to adversity [33], and children’s responses to stresses [34].

Identifying children at risk of negative outcomes as a result of exposure to parental depression offers the opportunity for early intervention [35]. In so doing, early interventions could help improve the long-term outcomes for children and potentially interrupt the intergenerational transmission risk of depression. By assessing those factors that have contributed to the development of resilience in children who have been exposed to postnatal depression, there may be a better understanding of those aspects of a child’s early environment that may help nurture and support them in withstanding the effects of postnatal depression.

Numerous studies have been conducted that provide evidence of the detrimental effect that postnatal depression can have on the developing child, both in the short- and the long-term [6]. Yet, few studies have looked at the mechanisms of parenting by which children exposed to maternal depression in the postnatal period may overcome adversity and may achieved normative behavioral development. Such studies are urgently needed.

The present study aims to develop a greater understanding of the maternal parenting practices and attitudes that foster resilience and normative behavioral development in children exposed to postnatal depression. In particular, we wished to identify those positive aspects of maternal parenting, such as interactive play and engagement that may protect infants against the adverse emotional and behavioral consequences of postnatal depression which may be amenable to change through intervention, and thus promote resilience.

## Methods

### Sample

The study is based on the Avon Longitudinal Study of Parents and Children (ALSPAC), a longitudinal, prospective cohort study of women, their partners and their children. All pregnant women with an estimated delivery date between 1 April 1991 and 31 December 1992, living in the former Avon Health Authority in England, were invited to participate in ALSPAC. The initial core sample consisted of 14,541 pregnancies, with 13,988 children alive at 1 year of age. All data was collected via postal questionnaires completed by the mother at different time points, and contained questions relating to herself and her child. Further information detailing sample recruitment, dropout, and methodology of ALSPAC is available elsewhere [36,37,38]. Please note that the study website contains details of all the data that is available through a fully searchable data dictionary (www.bris.ac.uk/alspac/researchers/data-access/datadictionary/). Ethical approval for the study was obtained from the ALSPAC Ethics and Law Committee and the Local Research Ethics Committees.

**Fig 1:**
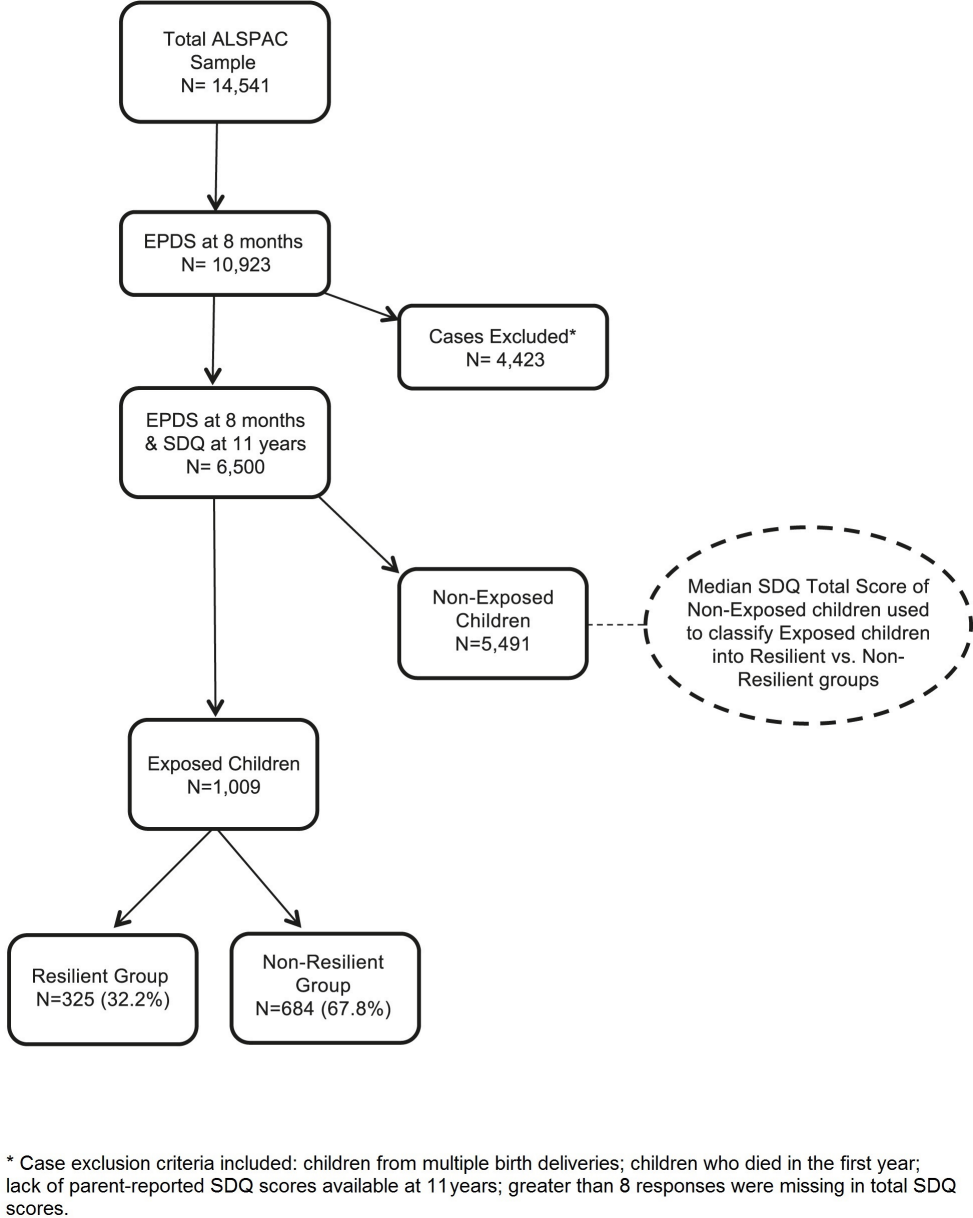
Flow chart showing sample derivation.

Information on depressive symptoms was available from scores on the Edinburgh Postnatal Depression Scale completed at 8 months postnatally by 10,293 mothers (76% of ALSPAC sample). From this subset, additional cases were excluded: children from multiple birth deliveries, and children who died in the first year. Further cases were excluded where we could not derive a total problem score on the parent-reported version of the Strengths and Difficulties Questionnaire completed at 11 years of age (either due to non-completion or else if more than 8 item responses were missing). This resulted in a final dataset of n = 6,500 (63%; see Fig 1).

### Measures

#### Maternal Postnatal Depression

Children’s exposure to maternal postnatal depression was assessed using the Edinburgh Postnatal Depression Scale (EPDS) [39]. The EPDS is a well-validated and extensively-used screening tool for postnatal depression, with a sensitivity of 85% and specificity of 82% [40]. It is a 10-item self-report questionnaire upon which women rate their feelings over the previous week, with a score ranging from 0 to 30. Scores of greater than 10 on the EPDS have sensitivity and specificity to detect clinically meaningful depression in a postnatal population [41]. Women completed the EPDS at 8 months postnatally, and were grouped as follows: a score below 10 on the EPDS indicated a likely absence of depression, and a score equal to or greater than 10 indicating possible presence.

EPDS scores at 8 months postnatally were used in the analyses. Although mothers were asked to rate their mood during the preceding week, evidence suggests that the rating is likely to reflect mood over a more extended period of time [42]. It is well-documented, including a study using the current sample, that women who meet criterion for depression later in the postpartum year were likely to have also met criteria for depression at earlier points in the perinatal period [30,43,44]. At 8 months of age children are increasingly aware of their surroundings from a social and emotional perspective and they engage in social referencing particularly with their mother [45]. In light of these factors, it was felt that this would be an optimal time to assess the child’s exposure to postnatal depression.

#### Child Development and Maternal Parenting Questionnaires

Mothers in ALSPAC were asked to complete questionnaires about their child as well as their role as a parent. We used questionnaire data collected at 5 time points within the first 24 months postnatally: four weeks, 8-, 15-, 18-, and 21-months. For this study, items reflecting a comprehensive range of key domains in child development were chosen: mother-infant relationship, maternal perceptions of parenting, negative and aggressive behaviors towards the child, child non-verbal communicative behavior and how the mother reported responding to her child’s emotional and practical needs.

A subset of items from the ALSPAC adaptation of the MacArthur Communicative Development Inventory (MCDI) [46] was used to assess the child’s non-verbal communication through actions and gestures that are not dependent upon verbal expression. A subset of items from the ALSPAC adaptation of the Home Observation for Measurement of the Environment (HOME) [47] was used to assess aspects of maternal engagement and obtain a better understanding of the ways the mother interacted with her child (see Table 1 for item examples).

**Table 1:** Variables retained in Confirmatory Factor analysis and Structural Equation Model.

Factor	Variable Name	Resilient (n = 325), Mean endorsement (sd)	Non-Resilient (n = 684), Mean endorsement (sd)
Maternal Engagement (18 months)	Mother teaches child songs (1 = yes, 2 = no)	0.92 (0.27)	0.91 (0.28)
	Mother teaches nursery rhymes(1 = yes, 2 = no)	0.95 (0.22)	0.92 (0.27)
	Mother sings to child (1–4: 1 = almost daily, 4 = never)	3.45 (0.9)	3.35 (1.00)
	Mother reads to child (1–4: 1 = almost daily, 4 = never)	3.6 (0.75)	3.47 (0.86)
	Mother plays games with child (1–4: 1 = almost daily, 4 = never)	3.64 (0.76)	3.58 (0.76)
	Mother has physical play with child (1–4: 1 = almost daily, 4 = never)	3.36 (0.92)	3.3 (0.99)
Negative Child Perception (4 weeks)	Baby is placid (1–5: 1 = very like, 5 = very unlike)	2.21 (1.1)	2.29 (1.17)
	Baby is grizzly (1–5: 1 = very like, 5 = very unlike)	3.72 (1.2)	3.54 (1.25)
	Baby is fretful (1–5: 1 = very like, 5 = very unlike)	3.92 (1.1)	3.82 (1.15)
	Baby is angry (1–5: 1 = very like, 5 = very unlike)	4.17 (0.96)	4.02 (1.06)
	Baby is stubborn (1–5: 1 = very like, 5 = very unlike)	3.65 (1.2)	3.48 (1.28)
Positive Child Perception (4 weeks)	Mother feels known by baby (1 = yes, 2 = not sure, 3 = no)	1.07 (0.29)	1.11 (0.34)
	Baby smiles (1 = yes, 2 = not sure, 3 = no)	2.11 (1.02)	1.95 (1.03)
	Baby laughs (1 = yes, 2 = not sure, 3 = no)	3.22 (1.15)	3.07 (1.31)
	Baby is communicative (1 = yes, 2 = not sure, 3 = no)	1.99 (0.81)	2.12 (0.89)
	Baby is sociable (1 = yes, 2 = not sure, 3 = no)	1.87 (0.82)	1.97 (0.85)
	Baby is happy (1 = yes, 2 = not sure, 3 = no)	1.3 (0.51)	1.3 (0.52)
Child Non-verbal Communication (15 months)	Child extends arm to show something held (1 = not yet, 2 = sometimes, 3 = often)	2.86 (0.36)	2.8 (0.45)
	Child points to interesting object (1 = not yet, 2 = sometimes, 3 = often)	2.92 (0.29)	2.86 (0.36)
	Child waves unprompted at someone (1 = not yet, 2 = sometimes, 3 = often)	2.89 (0.36)	2.79 (2.79)
	Child extends arms to be picked up (1 = not yet, 2 = sometimes, 3 = often)	2.84 (0.4)	2.76 (0.48)
	Child shakes head ‘no’ (1 = not yet, 2 = sometimes, 3 = often)	2.9 (0.32)	2.87 (0.40)
	Child nods head ‘yes’ (1 = not yet, 2 = sometimes, 3 = often)	2.54 (0.75)	2.51(0.75)
	Child puts finger to lips to hush (1 = not yet, 2 = sometimes, 3 = often)	1.95 (0.92)	1.84 (0.88)
	Child opens & closes hand to ask for item (1 = not yet, 2 = sometimes, 3 = often)	1.38 (0.7)	1.36 (0.70)
	Child blows kiss from a distance (1 = not yet, 2 = sometimes, 3 = often)	2.13 (0.83)	2.16 (0.83)
Positive Views of Parenting (8 months & 21 months)	Mother feel her baby is fun (1–4: 1 = exact feeling, 4 = never feels)	1.7 (0.76)	1.58 (0.80)
	Mother feels fulfilled by baby (1–4: 1 = exact feeling, 4 = never feels)	2.12 (0.94)	1.84 (0.78)
	Mother takes pleasure in baby’s development (1–4: 1 = exact feeling, 4 = never feels)	1.17 (0.44)	2.3 (0.98)
	Mother enjoys her baby (1–4: 1 = exact feeling, 4 = never feels)	1.63 (0.64)	1.21 (0.50)
	Mother feels child gives her great joy (1–4: 1 = exact feeling, 4 = never feels)	1.17 (0.44)	1.64 (0.65)
	Mother feels pleasure watching her child grow (1–4:1 = exact feeling, 4 = never feels)	1.12 (0.35)	1.28 (0.56)
	Mother feels glad she had child when she did (1–4:1 = exact feeling, 4 = never feels)	1.24 (0.6)	1.28 (0.57)
	Mother really loves her child (1–4:1 = exact feeling, 4 = never feels)	1.06 (0.25)	1.36 (0.69)
	Mother feels her child is fun (1–4:1 = exact feeling, 4 = never feels)	1.65 (0.65)	1.11 (0.38)

#### Child Psychological Functioning

Children’s psychological functioning at 11 years was assessed using the Strengths and Difficulties Questionnaire (SDQ) [48], an instrument for three to 16 year olds that screens for behavior and emotional problems. The scale comprises 25 items across 5 subscales (emotional problems, hyperactivity, conduct problems, peer problems, and a prosocial score). The first four subscales can be combined to derive a total difficulties score where higher scores indicate more problem behavior. We therefore chose to use the SDQ total difficulties score rather than subscale scores, as this score of the SDQ has been used extensively and has demonstrated reliability and validity, with good internal consistency (Cronbach’s alpha = 0.82) and re-test stability (mean = 0.72) [49].

The outcome at 11 years was chosen because the children were in the latter part of childhood and prior to adolescence which is a noted time of behavioral and emotional change. Scores were pro-rated where responses were missing, and cases were only included if fewer than 8 responses were missing in the calculation of the total difficulties score. We looked at one time point in childhood rather than several as the stability over time of resilience remains to be established. Ultimately, we wanted to assess typical behavior (in relation to the child’s peers) in a group of children who have been exposed to unusual maternal behavior that would ordinarily predispose them to negative behavioral outcomes.

In order to investigate differences in the long-term response to adversity, children exposed to maternal postnatal depression during the first year were compared according to their behavioral outcome at 11 years of age. Similar to the method used by Jaffee et al.[50], we have employed a reference point requiring functioning that is at or above the median of the sample of non-exposed ALSPAC children who provide a marker of behavioral functioning in children not exposed to maternal depression in their postnatal environment.

Children whose mothers experienced high depressive symptom scores at 8 months postnatally were allocated to the ‘exposed’ group (n = 1,009; 16%). Children whose mothers had low depressive symptom scores at 8 months were allocated to the ‘non-exposed’ group (n = 5,491; 84%; see Fig 1).

The median SDQ total difficulties score of the ‘non-exposed’ group was used to establish the cut-off point for resilience in the ‘exposed group’. Children in the exposed group whose total difficulties score at age 11 was at or below the median score for the ‘non-exposed’ group were allocated to the ‘resilient’ group (n = 325; 32%). Children in the ‘exposed’ group with scores that were above the median cut-off were allocated to the ‘non-resilient’ group (n = 684; 68%; see Fig 1). As severity of depression may account for apparent differences in resilience, this was adjusted for in the main analyses.

### Statistical Analysis

Descriptive statistics and frequencies were used to examine the study population, and were performed in STATA version 12 [51]. A structural equation modeling (SEM) approach was chosen to establish the mechanisms underlying resilience as it provides a more robust and reliable alternative to classical methods for investigating relationships between observed variables and latent factors. Exploratory (EFA) and Confirmatory factor analyses (CFA) as well as SEM were conducted using Mplus version 7 [52].

Items from the first round of qualitative review of the maternal parenting and child development questionnaires were subjected to exploratory factor analysis (EFA). The resulting EFA solutions were reviewed, taking care to adhere to the recommended criteria including an assessment of eigenvalues, review of model fit statistics, and ensuring factor loadings were salient at greater than 0.4 [53,54].

Due to the categorical nature of the data, and to account for missing data, the models were estimated using robust weighted least squares means and variance adjusted estimator (WLSMV) [55,53]. The fit of the models were assessed using the comparative fit index (CFI), the Tucker-Lewis index (TLI), and the root mean square error of approximation (RMSEA). According to Brown [53] and Hu and Bentler [56], the following guidelines are suggested for the evaluation of model goodness-of-fit: 1) significant *Χ* ^2^ statistic; 2) RMSEA < 0.06; and 3) CFI and TLI > 0.95. These values are not absolute criterion as they are subject to fluctuations of modeling conditions. Thus, model fit statistics are interpreted in relation to the theoretical constructs under investigation.

## Results

Demographic characteristics of the main sample used in analyses are summarized in Table 2. From the subset of 6,500 participants, 1,009 women had an EPDS score of greater than 9 (mean = 10.6; s.d. = 4.9), and were categorized as having higher levels of depressive symptoms indicating possible depressive disorder at 8 months postnatally. Severity of maternal depressive symptoms was adjusted in the analyses for the resilient (mean EPDS score = 9.7; s.d. = 5.2) and non-resilient groups (mean EPDS score = 10.97; s.d. = 4.9).

**Table 2:** Sample Characteristics.

		Resilient (Exposed, Low SDQ; n = 325), *n* (%)	Non-Resilient (Exposed, High SDQ; n = 684), *n* (%)
**Child Gender**	Male	151 (46.5%)	360 (52.5%)
	Female	174 (53.5%)	324 (47.5%)
**Maternal Education**	CSE & Vocational	79 (24.3%)	186 (26.8%)
	O Level	105 (32.3%)	219 (32%)
	A Level	83 (25.5%)	171 (25%)
	Degree	55 (16.9%)	91 (13.3%)
**Marital Status**	Never Married	41 (12.6%)	104 (15.2%)
	Widowed	1 (<0.1%)	2 (<0.1%)
	Divorced	17 (0.1%)	43 (0.1%)
	Separated	8 (<0.1%)	12 (<0.1%)
	1^st^ Marriage	235 (72.3%)	469 (69%)
	Marriage 2 or 3	21 (0.1%)	46 (0.1%)

SDQ total difficulties scores at 11 years of age were assessed (mean = 8.78; s.d. = 5.7), and compared between groups of exposed children. Of those children whose mothers had high scores on depressive symptoms, the resilient group had a lower mean score (3.2) than the non-resilient group (11.4; difference = 8.2; 95% CI = 7.8–8.6; p<0.001).

Table 3 provides demographic details of the ALSPAC mothers who did not experience depressive symptoms at or above a score of 10 on the EPDS at 32 weeks and those mothers of children without SDQ scores at 11 years. These data are provided to illustrate how similar the characteristics are for both maternal groups.

**Table 3:** Characteristics of ALSPAC mothers without depressive symptoms vs. mothers with depressive symptoms included in current analyses(*Children of these mothers form part of the exposed group if SDQ scores are available at 11 years of age.)

		Mothers without depressive symptoms, *n* (%)	Mothers with depressive symptoms*, *n* (%)
**Maternal Age**	N_(total)_ = 10,293	N = 8,991	N = 1,932
	<16–20	432 (4.8%)	137 (7.1%)
	21–26	2,625 (29.1%)	589 (30.0%)
	27–32	4,199 (46.7%)	833 (43.1%)
	33–38	1,547 (17.2%)	325 (16.8%)
	39 - >43	188 (2.1%)	48 (2.4%)
**Maternal Education**	N_(total)_ = 10,548	N = 8,707	N = 1,841
	CSE & Vocational	2,226 (25.6%)	628 (34.1%)
	O Level	3,143 (36.1%)	585 (31.8%)
	A Level	2,073 (23.8%)	401 (21.8%)
	Degree	1,230 (14.1%)	211 (11.5%)
	Missing	35 (0.4%)	16 (0.8%)
**Marital Status**	N_(total)_ = 10,697	N = 8,820	N = 1,877
	Never Married	1,364 (15.5%)	377 (20.1%)
	Widowed	11 (0.1%)	3 (0.2%)
	Divorced	317 (3.6%)	107 (5.7%)
	Separated	108 (1.2%)	50 (2.7%)
	1^st^ Marriage	6,386 (72.4%)	1,200 (63.9%)
	Marriage 2 or 3	569 (6.5%)	128 (6.8%)
	Missing	65 (0.7%)	12 (0.6%)
**Parity**	N_(total)_ = 10,654	N = 8,793	N = 1,861
	0	4,028 (45.8%)	704 (37.8%)
	1	2,973 (33.8%)	719 (38.6%)
	2	1,232 (14.0%)	269 (14.6%)
	3–13	429 (4.9%)	129 (6.9%)
	Missing	131 (1.5%)	40 (2.1%)

**Fig 2:**
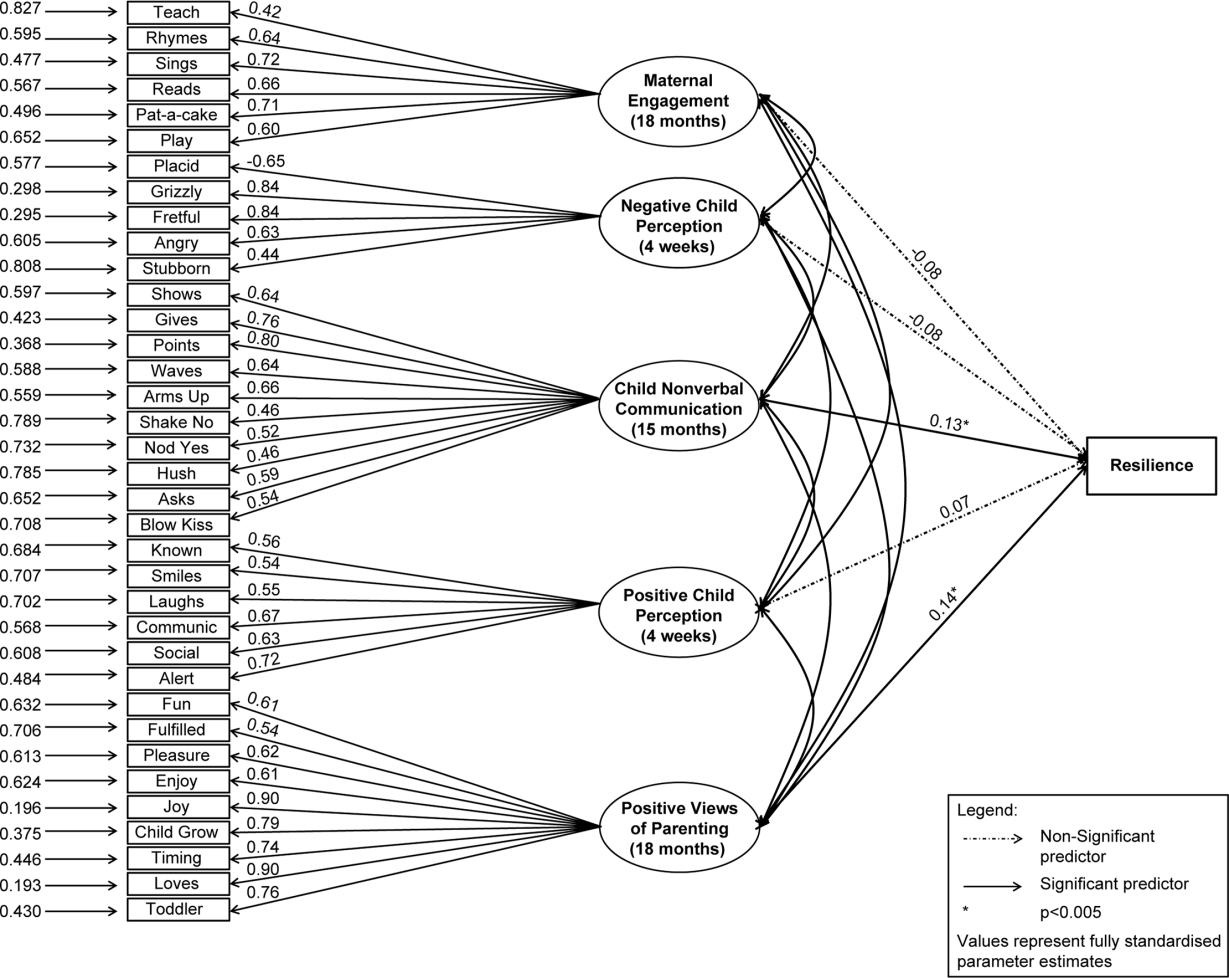
Model diagram.

The optimal Exploratory Factor Analysis solution of the parenting and child communication variables revealed five latent factors: *Maternal Engagement*, *Negative Child Perception*, *Positive Child Perception*, *Positive Views of Parenting*, and *Nonverbal Child Communication* (see Fig 2). These variables were then tested in a five factor Confirmatory Factor Analysis model with *Total Difficulties* for the child as the outcome variable. Based on overall model fit and individual variable factor loadings according to parameters details above, the CFA model was evaluated. Items retained in the final CFA model are shown in Table 1. The model was over-identified with 600 df; Χ^2^ (600) = 816.44, *p*< 0.01. The statistics suggested good model fit: CFI = 0.98, TLI = 0.98, RMSEA = 0.02. Fully standardized estimates indicate that three of the factors were not predictors of resilience: *Maternal Engagement* with appropriate child centred activities like singing and reading stories (β = -0.08, *p* = 0.20), *Negative Child Perception* (β = -0.08, *p* = 0.13), and *Positive Perception* of the child (β = 0.07, *p* = 0.26) reflected in both critical attributions of the baby. In contrast *Positive Views of Parenting*, with items reflecting a strong emotional attachment to and enjoyment of her baby in the role as a parent was a predictor of the outcome variable ‘resilience’ (β = 0.14, *p*<0.05), indicating that the more positive a mother is about being a mother, the greater likelihood her child was resilient to the exposure of symptoms of maternal postnatal depression and show less in terms of later behavioural problems. Further, *Nonverbal Child Communication* was also a predictor of resilience (β = 0.13, *p*<0.05), suggesting that the more the child communicates effectively in a non-verbal way, the more likely they were to attain normative behavioral development.

## Discussion

The primary objective of this study was to gain a better understanding of the mechanisms of resilience in children exposed to postnatal depression. Specifically, we wanted to investigate the aspects of maternal parenting that were involved in the development of resilience and might be amenable to intervention in populations of women with postnatal depression.

Using a large, cohort study, we provide evidence that a mother’s positive perspective about her parenting role contributes to the child’s development of resilience to the adverse effects of postnatal depression, thereby providing a protective effect. Prior evidence regarding parenting has emphasized that depressed women display less-optimal parenting abilities, less sensitive modes of responding, and are more punitive and rejecting towards their infants [57,9,10]. Unsurprisingly, studies report depressed mothers feeling less efficacious in their parenting role than do non-depressed mothers [15]. While we did not explicitly examine the mother’s negative perspective of her parenting role, this is worthy of further investigation as it could be a contributing factor in non-optimal child development. As described by Bandura [58], among the mechanisms of human agency, there is none as central or profound as an individual’s belief in their ability and efficacy to exercise control over their own lives and actions.

Despite earlier findings, the current study illustrates that the degree of positive feelings about parenting within a population of postnatal women with elevated depressive symptomatology lends itself to fostering the resilience in their child. It supports similar findings by Jones et al. [59], who investigated the protective role of family cohesion and parenting against the effects of common stressful events in childhood (such as moving house, parental smoking, etc.). They found that positive parenting behaviors and interactions with their child were associated with better verbal skills and fewer behavioral difficulties at a younger age in their study of the ALSPAC cohort (age 7 years compared with 11 years in the current study). This confirms speculations made previously by Brennan et al. [60], suggesting that positive maternal parenting characteristics may have important protective effects in high-risk populations, serving to neutralize some of the effects of the risks encountered.

A further finding of the current study was that children who, at 15 months, are more able to communicate in a non-verbal way have a greater likelihood of being resilient at 11 years. Previous studies have shown that positive temperamental features of the child are more likely to elicit warm responses from those around them [11]. Due to the nature of the data collection in the ALSPAC sample, it is not clear whether the non-verbal communication displayed by the children at age two elicited warm responses from their mothers. However, it could be speculated that such non-verbal forms of communication might lead to positive chains of responses from the mother, which in turn fostered resilience [61]. Further, it could be the case that maternal positive feelings about parenting led to more nonverbal communication from the child [62]. Conversely, those children with lower non-verbal means of communicating could have been displaying early signs of developmental difficulties unrelated to their mothers’ depressed symptomatology. In their own right, these difficulties may have contributed to the interruption in the flow of communication between mother and infant. These issues are worthy of further investigation.

Our results suggest aspects of maternal parenting within the first two years of a child’s life that offer opportunities for intervention. Targeting aspects of parenting that support maternal role development, positive mother-child interaction and communication may prove a useful method of increasing the likelihood of resilient outcomes in children. It is widely acknowledged, and increasingly advocated, that early childhood is an optimal window of opportunity to ensure that children have the necessary resources available to be well-prepared to embark on development [63]. The early years of life are a period of maximal brain development and learning when the foundations of cognitive, social, and emotional development are established [64].

There are some limitations of this study. The EPDS was designed to screen for depressive symptoms, and is not meant to be used as a standalone diagnostic instrument for depressive illness. However, scores of 10 and above have been shown to be highly correlated with observer-rated diagnostic criteria for depression [65]. The measures used were based solely on maternal reports, raising the possibility of shared method variance. Further, the reports were from mothers with depression of varying degrees of severity which might have affected the accuracy of their self-reports [66,67]. This may not have had a considerable effect, as it has been suggested that mothers with depression can provide sufficiently accurate accounts of their child’s behavior [68]. The ALSPAC sample is not representative of the UK population as a whole, and further, the sub-samples used in this study may not be representative of the cohort as a whole. Generalizability of results may therefore be limited. The findings of the current factor model warrants replication with more socioeconomically and ethnically diverse samples. As with many large longitudinal cohort studies, attrition of the sample over time may have influenced the results. Table 2 illustrates that the sample of mothers not included in the analyses because of a lack of significant depressive symptoms or missing SDQ scores for their child suggests that, for the ALSPAC sample, both included and excluded cases are characteristically similar. It is important to note that an earlier study of the ALSPAC cohort found that women with mental health problems were less likely to return the questionnaires at each wave of data collection than those women without self-reported anxiety and depression [44].

Maternal depression is associated with a number of other family- and community-based adversities which can affect child outcomes [4]. Assessing the impact of such risks and potential opportunities for resilience could be considered in future studies. It is becoming increasingly evident that the role of the father or partner is instrumental in child development. The quality of the partner relationship and involvement are crucial in the child’s development of coping capacities. When the child is facing adversity that originates with one parent, the quality of relationship and supportive competence with the other family members and the wider socio-economic environment is of critical importance [10]. Work needs to be conducted to determine if similar aspects of paternal parenting (such as paternal positive feelings about parenting) can help serve a protective role for a child facing the adversity of depression at an early age, with a view to a more family-focused approach to understanding the issues. Programs emphasizing a family-focused approach have shown promising outcomes [69,70].

Finally, the self-report questionnaires do not provide insight into why it is that some mothers were still able to remain positive in their perceptions of their own parenting, despite suffering from depression. Further research would benefit from a greater understanding of the origins of positive thinking and positive self-efficacy in mothers.

Maternal depression in the postnatal period can be viewed as an important public health concern due to the risk of adverse outcomes for the child in their early years, adolescence, and into adulthood, including the risk of developing depression themselves. However, as this study has illustrated, for some children their behavior and emotional state appear to be relatively unaffected despite exposure to depression; and this occurs through the mechanisms of positive parenting and children’s own non-verbal communicative skills.

In addressing the needs of children of mothers with postnatal depression and looking towards those aspects that could be amenable to change, it would appear to us that there are a number of elements, such as facilitating adjustment to and enjoyment in early parenting and supporting maternal positive feelings of parenting, that could help foster the child’s resilience and increase the likelihood of normal development.

Using the growing knowledge on resilience may be vital in guiding social policies for the promotion of wellbeing and positive adaptation across populations. In so doing, early interventions could help improve the long-term outcomes for children as well as potentially interrupt the intergenerational transmission risk of depression. By assessing those factors that have contributed to the development of resilience in children who have been exposed to postnatal depression, there is a better understanding of those aspects of a child’s early environment that may help nourish and support them to withstand the effects of the symptoms of postnatal depression.
